# An Arabidopsis *PTH2* Gene Is Responsible for Gravity Resistance Supporting Plant Growth under Different Gravity Conditions

**DOI:** 10.3390/life12101603

**Published:** 2022-10-14

**Authors:** Takayuki Hattori, Kouichi Soga, Kazuyuki Wakabayashi, Takayuki Hoson

**Affiliations:** 1 Department of Biology, Graduate School of Science, Osaka City University, Sumiyoshi-ku, Osaka 558-8585, Japan; 2 Department of Biology, Graduate School of Science, Osaka Metropolitan University, Sumiyoshi-ku, Osaka 558-8585, Japan

**Keywords:** Arabidopsis (*Arabidopsis thaliana*), cell wall, cortical microtubules, elongation growth, gravity resistance, hypergravity, lateral growth, mechanical properties, *PEPTIDYL-tRNA HYDROLASE II* (*PTH2*)

## Abstract

Terrestrial plants respond to and resist gravitational force. The response is termed “gravity resistance”, and centrifugal hypergravity conditions are efficient for investigating its nature and mechanism. A functional screening of Arabidopsis T-DNA insertion lines for the suppression rate of elongation growth of hypocotyls under hypergravity conditions was performed in this study to identify the genes required for gravity resistance. As a result, we identified *PEPTIDYL-tRNA HYDROLASE II* (*PTH2*). In the wild type, elongation growth was suppressed by hypergravity, but this did not happen in the *pth2* mutant. Lateral growth, dynamics of cortical microtubules, mechanical properties of cell walls, or cell wall thickness were also not affected by hypergravity in the *pth2* mutant. In other words, the *pth2* mutant did not show any significant hypergravity responses. However, the gravitropic curvature of hypocotyls of the *pth2* mutant was almost equal to that of the wild type, indicating that the *PTH2* gene is not required for gravitropism. It is suggested by these results that *PTH2* is responsible for the critical processes of gravity resistance in Arabidopsis hypocotyls.

## 1. Introduction

Terrestrial plants have been constantly exposed to gravity on Earth since the aquatic ancestors of terrestrial plants landed during their evolution [1,2]. Since then, terrestrial plants have developed a sequence of adaptations to survive at 1 *g* and have used gravity as a signal to regulate growth and development. Gravitropism is defined as the directional growth response of plants in response to the direction of gravity and is recognized as a primary graviresponse in plants [3]. Mechanical resistance to the gravitational force (gravity resistance) is the principal graviresponse in plants in addition to gravitropism [4,5]. Conditions that facilitate the analysis of the mechanisms in gravity resistance but are difficult to duplicate on Earth are provided by true microgravity. Therefore, the nature and mechanisms of gravity resistance have mainly been studied using basipetal hypergravity produced by centrifugation [4,5].

Hypergravity generally suppresses elongation growth but stimulates the lateral growth of the stem organs of plants [6,7,8,9,10,11,12]. In other words, plants have short and thick bodies under hypergravity conditions. The construction of a short and thick body is considered an essential factor in gravity resistance. The shape of the plant body is predominantly determined by the direction of the expansion of individual cells. Cortical microtubules, a characteristic structure in the interphase cells of plants, are responsible for regulating the direction of cell expansion [13,14]. The frequency of cells having longitudinal microtubules in the epidermal cells of azuki bean epicotyls [15] and Arabidopsis hypocotyls [16,17] is increased by hypergravity. The reorganization of cortical microtubules was also accelerated by hypergravity in protoplasts from *Brassica* *napus* hypocotyls [18]. The modification of the dynamics of cortical microtubules required for the regulation of the direction of cell expansion in response to hypergravity stimuli is suggested by these results.

Plant cells are enclosed by highly developed cell walls, which provide their cells with structural support and mechanical strength. Thus, the cell wall may play a significant role in gravity resistance, analogous to the role of bones and muscles in an animal body. Previously, we illustrated that cell wall extensibility in various plant materials is decreased by hypergravity [8,9,10,11,19]. Namely, cell wall rigidity increases, resulting in a tough body under hypergravity conditions. Increasing the cell wall rigidity and constructing a short and thick body are considered essential factors for gravity resistance. Cellulose and matrix polysaccharides, such as pectin and hemicellulose, are the major components of plant cell walls. Cell wall thickness is one of the factors determining cell wall extensibility. The levels of cell wall polysaccharides, such as cellulose, per unit length of the shoot were increased by hypergravity in various plant materials [8,9,10,11,12]. Namely, cell wall thickness is increased under hypergravity conditions, leading to a decrease in cell wall extensibility.

We performed a functional screening of confirmed homozygous T-DNA insertion lines in Arabidopsis produced in the SALK institute (the Salk Unimutant Collection) project for the suppression rate of elongation growth of hypocotyls under hypergravity conditions to identify the genes required for gravity resistance. These lines were successfully used to specify a novel cell wall-related gene, namely *ANTHOCYANINLESS2* [20]. As a result, we identified *PEPTIDYL-tRNA HYDROLASE II* (*PTH2*), a gene required for gravity resistance. *PTH2* encodes an enzyme that releases tRNA from peptidyl-tRNA by cleaving the ester bond between the peptide and the tRNA [21]. Changes in growth, the orientation of cortical microtubules, the mechanical properties of cell walls, and the levels of cell wall polysaccharides were examined in the *pth2* mutant to clarify the roles of *PTH2* in gravity resistance. We also examined the gravitropic curvature in the *pth2* mutant to confirm whether *PTH2* is required not only for gravity resistance but also for gravitropism.

## 2. Materials and Methods

### 2.1. Plant Materials and Growth Analysis

Wild-type *Arabidopsis thaliana* (L.) Heynh. (ecotype Columbia-0) and confirmed homozygous T-DNA insertion lines of Arabidopsis (Arabidopsis Biological Resource Center, Columbus, OH, USA) were used in this study. The plant materials were prepared as previously described [22]. For hypergravity treatment, the seedlings were exposed to basipetal hypergravity at 300 *g* with a centrifuge (H-28-F; Kokusan, Tokyo, Japan) at 25 °C for 24 h in the dark. Hypocotyl length was measured using a scale. Epidermal cells of hypocotyls were observed with a scanning electron microscope (Miniscope TM-1000; Hitachi, Tokyo, Japan). The length and width of the cells were measured using ImageJ software (http://rsbweb.nih.gov/ij, accessed on 1 October 2022; NIH).

Arabidopsis seedlings were cultivated for 48 h, and the seedlings were transferred on the surface of a 0.8% (*w*/*v*) agar medium in a plastic Petri dish to analyze the gravitropic curvature. The curvature was measured after rotating a plastic Petri dish containing vertically fixed seedlings at 90° and at 25 °C for 24 h in the dark. After treatment, the curvature of the hypocotyls was measured using ImageJ software.

### 2.2. Immunofluorescence Microscopy

Cortical microtubules were observed by immunofluorescence microscopy, as described previously [16]. Primary antibodies against α-tubulin (product T6199; Sigma-Aldrich, Saint Louis, MO, USA) and a secondary antibody, Cy3-conjugated anti-mouse IgG (product C2181; Sigma-Aldrich), were used for the visualization of cortical microtubules. Immunofluorescence images were obtained with a fluorescence microscope (Axio Imager. A1; Carl Zeiss, Göttingen, Germany) equipped with a cooled CCD camera (DP74; Olympus, Tokyo, Japan) and processed with bundled image processing software (cellSens Imaging Software; Olympus). As for the orientation of cortical microtubules, the frequency of cells with cortical microtubules within a range of 0–20° (longitudinal), 20–70° (oblique), and 70–90° (transverse) to the longitudinal cell axis, and in a variety of directions (random), was determined.

### 2.3. Measurement of the Mechanical Properties of Cell Walls

Samples for measuring the mechanical properties of the cell walls were prepared as described previously [20]. The cell wall extensibility and breaking load of the hypocotyls were measured using a tensile tester (Tensilon STB-1225S; A&D, Tokyo, Japan). Hypocotyls were fixed between two clamps at a distance of 0.5 mm and stretched by raising the upper clamp at a rate of 20 mm min^−1^ until the hypocotyls were broken. The cell wall extensibility (strain load^−1^, μm g^−1^) was determined by measuring the load’s rate of increase from 0.8 g to 1.0 g.

### 2.4. Quantification of Cell Wall Polysaccharides

As described previously, the fractionation and quantification of cell wall polysaccharides were performed [20]. Hypocotyls excised from seedlings (120–220 per batch) were used for the analysis. Pectin substances were extracted with 50 mM EDTA at 100 °C. Hemicellulose I and hemicellulose II were extracted with 4% (*w*/*v*) KOH and 24% (*w*/*v*) KOH containing 0.02% NaBH_4_ at 25 °C, respectively. The alkali-insoluble fraction was designated as cellulose. The total sugar content of each fraction was determined using the phenol-sulfate method [23] and expressed as glucose equivalents.

## 3. Results

### 3.1. Identification of PTH2 as a Gene Required for Gravity Resistance

Hypergravity at 300 *g* suppressed the elongation growth of hypocotyls by 20% in the wild type. We performed a functional screening of Arabidopsis T-DNA insertion lines for the suppression rate of hypocotyl elongation at 300 *g* to identify the genes required for gravity resistance. As a result, we obtained several lines whose suppression rates were modified from those of the wild type. The growth analysis was repeated at least three times with the reproduced seeds for the selected lines to confirm the modifications.

**Figure 1:**
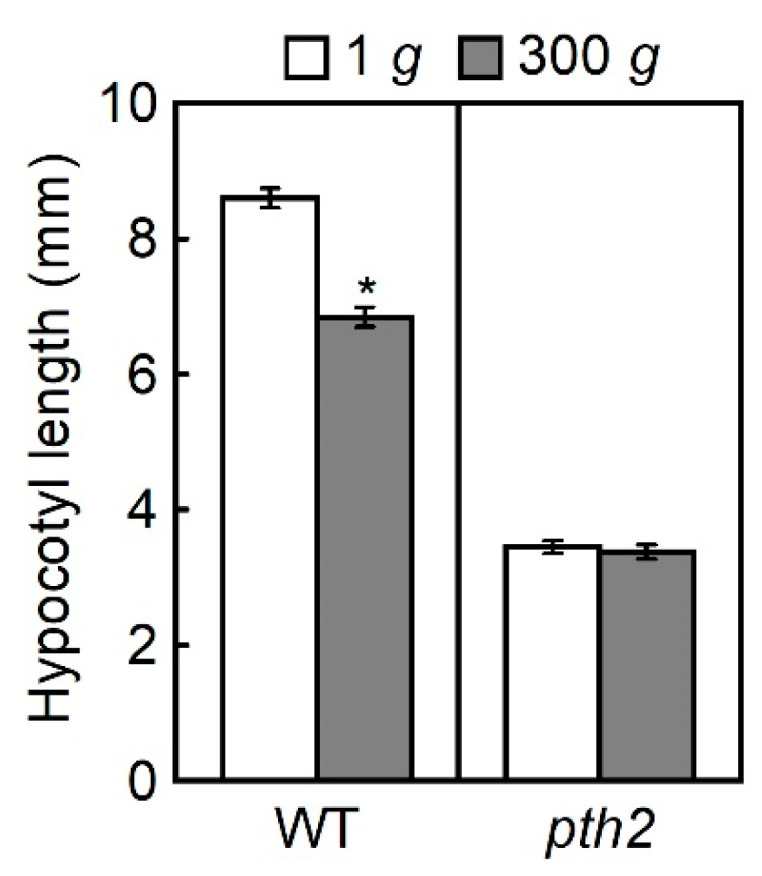
The length of the hypocotyls at 300 *g*. Values are the means ± SE (*n* = 20). WT, wild type. * Mean value was significantly different between 1 *g* and 300 *g* (Student’s *t*-test: *p* < 0.05).

One confirmed gene was *PEPTIDYL-tRNA HYDROLASE II* (*PTH2*; At4g32900, SALK_048173C). Hypergravity suppressed hypocotyl elongation in the wild type but did not affect that of the *pth2* mutant (Figure 1). The length of etiolated hypocotyls in the *pth2* mutant was about 60% shorter than in the wild type at 1 *g*. Growth suppression by hypergravity might not be detected due to the low growth rate of the *pth2* mutant. We examined the elongation growth of the *pth2* mutant in the presence of salt stress (50 mM NaCl) to assess this possibility and found that elongation growth was suppressed to the same extent as in the wild type (data not shown). It was indicated by these results that the suppression of elongation growth could be detected even at low growth rates in the *pth2* mutant.

**Figure 2:**
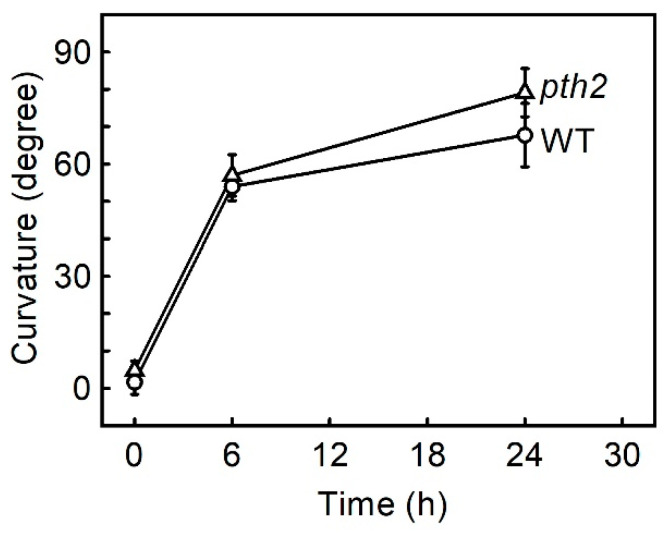
Gravitropic curvature in hypocotyls. Values are the means ± SE (*n* = 14–15). WT, wild type.

The gravitropic curvature of the *pth2* mutant is shown in Figure 2. The hypocotyl of the *pth2* mutant was bent in the direction opposite gravity at a similar rate to the wild type. This result indicates that the *PTH2* gene is not required to induce gravitropism.

### 3.2. Modification of Growth Anisotropy under Hypergravity Conditions

**Figure 3:**
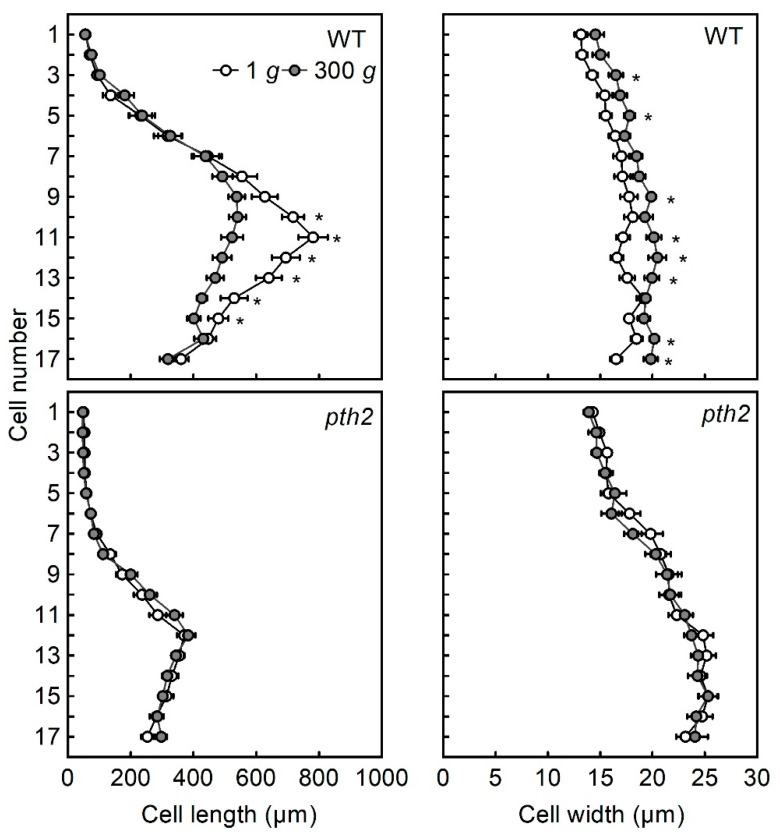
The length and width of epidermal cell files from the tip (cell 1) to the base (cell 17). Values are the means ± SE (*n* = 20). WT, wild type. * Mean value was significantly different between 1 *g* and 300 *g* (Student’s *t*-test: *p* < 0.05).

The length (left panel) and width (right panel) of individual epidermal cells within a cell file are shown in Figure 3. The epidermal cell files of the wild type and the *pth2* mutant hypocotyls consisted of about 17 cells. The frequency of cell division in the *pth2* mutant was indicated by this result to be similar to that of the wild type. Nonetheless, the hypocotyl length of the mutant was shorter than that of the wild type. At 1 *g*, the length of epidermal cells in the wild type increased rapidly from the tip to the 11th cell, but in the *pth2* mutant it was almost the same from the tip to the 7th cell. Comparing the length of individual epidermal cells of the wild type and of the *pth2* mutant in hypocotyls grown at 1 *g*, the length of the *pth2* cells was shorter than that of the wild type. In the wild type, the length of cells 10 to 15 was decreased by hypergravity. On the other hand, the length of the cells in the *pth2* mutant was not affected by hypergravity. In 1 *g*-grown hypocotyls, the width of wild-type cells was almost the same between tip and base, but the width of cells gradually increased from tip to base in the *pth2* mutant. Some cells in the wild type had an increased width due to hypergravity, but the width of the cells in the *pth2* mutant was not affected by hypergravity. Therefore, in the wild type, the cell shape was thickened and shortened by hypergravity, but there were no effects on the cell shape in the *pth2* mutant caused by hypergravity.

**Figure 4:**
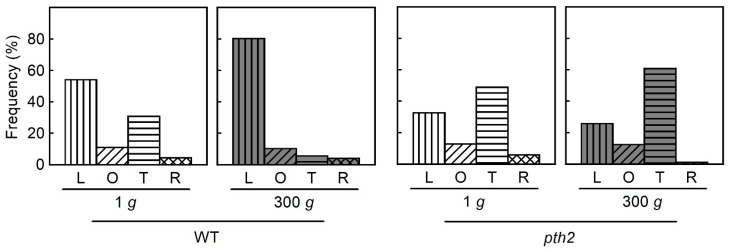
The orientation of cortical microtubules in epidermal cells of hypocotyls. WT, wild type. L, longitudinal (0–20°). O, oblique (20–70°). T, transverse (70–90°). R, random. *n* = 147–202.

The orientation of cortical microtubules adjacent to the outer tangential wall of epidermal cells was nearly uniform in individual cells. Thus, the cells were categorized into four types based on the orientation of the microtubules (longitudinal, oblique, transverse, and random) in the middle region (cells 10–12 in Figure 3) of hypocotyls (Figure 4). Cells with longitudinal microtubules were predominant in the wild type, regardless of gravitational conditions. On the other hand, in the *pth2* mutant, cells with transverse microtubules were predominant, regardless of gravitational conditions. Hypergravity decreased the cells with transverse microtubules and increased the cells with longitudinal microtubules in the wild type. However, the microtubule orientation in the *pth2* mutant was not affected by hypergravity.

### 3.3. Modification of Cell Wall Properties under Hypergravity Conditions

**Figure 5:**
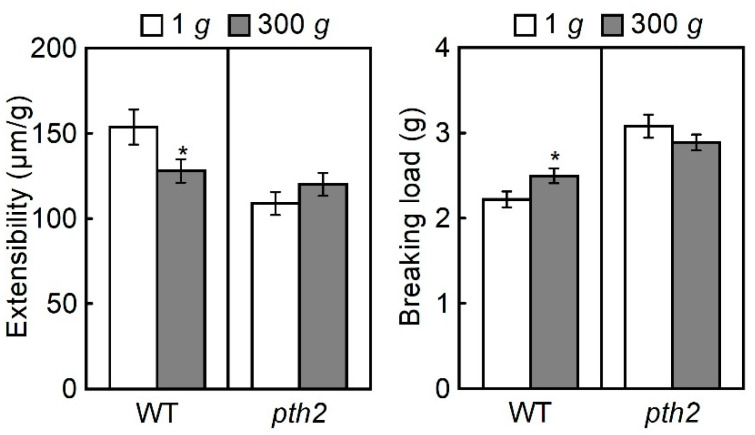
Cell wall extensibility and the breaking load of hypocotyls. Values are the means ± SE (*n* = 20). WT, wild type. * Mean value was significantly different between 1 *g* and 300 *g* (Student’s *t*-test: *p* < 0.05).

The cell wall extensibility (left panel) and the breaking load (right panel) of the hypocotyls measured using a tensile tester are shown in Figure 5. Comparing the cell wall extensibility of the wild type and the *pth2* mutant grown at 1 *g*, the cell extensibility of the *pth2* mutant was smaller than that of the wild type. On the other hand, the breaking load of the *pth2* mutant was larger than that of the wild type. It was indicated by these results that the cell walls of the *pth2* mutant are less extensible than those of the wild type, suggesting that hypocotyls of the *pth2* mutant are shorter because the cell walls are less extensible. The cell wall extensibility was decreased by hypergravity in the wild type while breaking load was increased. This is an indication that cell walls are made mechanically rigid in hypocotyls of the wild type by hypergravity. However, neither the cell wall extensibility nor the breaking load in the hypocotyls of the *pth2* mutant were affected by hypergravity.

**Figure 6:**
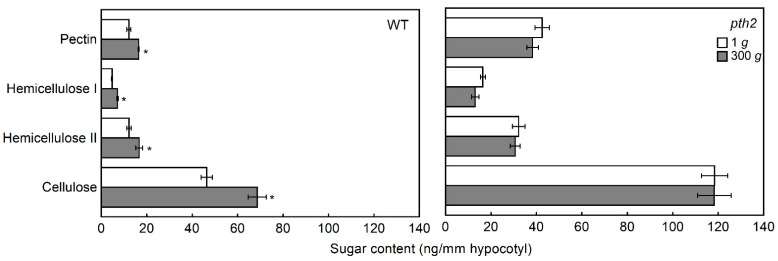
Amounts of cell wall polysaccharides per unit length of the hypocotyl. Values are the means ± SE (*n* = 4). WT, wild type. * Mean value was significantly different between 1 *g* and 300 *g* (Student’s *t*-test: *p* < 0.05).

Quantitative changes in the polysaccharides of cell walls may be the mechanism responsible for changes in cell wall extensibility and breaking load. The quantities per unit length of the hypocotyl in the pectin, hemicellulose I, hemicellulose II, and cellulose fractions are shown in Figure 6. Comparing the quantities of the wild type and the *pth2* mutant grown at 1 *g*, the quantities of all four fractions of the *pth2* mutant were larger than those of the wild type. It was indicated in these results that the cell wall thickness of the *pth2* mutant was greater than that of the wild type. The less extensible cell walls of the *pth2* mutant may be due to the thickness of the cell walls (Figure 5 and Figure 6). The quantities of all four fractions were increased by hypergravity in the wild type, indicating that the cell wall thickness was increased by hypergravity. However, the quantities of all four fractions in the *pth2* mutant were not affected by hypergravity.

## 4. Discussion

The functional screening of Arabidopsis T-DNA insertion lines for the suppression rate of elongation growth of hypocotyls under hypergravity conditions was performed in this study to identify the genes required for gravity resistance. In the wild type grown under hypergravity conditions at 300 *g*, hypocotyl elongation was suppressed by 20%. However, hypergravity did not affect elongation growth in the T-DNA insertion line of the *PTH2* gene isolated in this study (Figure 1). It has been shown that hypergravity not only suppresses elongation growth, but also stimulates lateral growth in the stem organs of various plants [6,7,8,9,10,11,12]. Namely, hypergravity modifies growth anisotropy in the cells of stem organs. Hence, we investigated whether the hypergravity-induced modification of growth anisotropy occurred in the epidermal cells of hypocotyls in the *pth2* mutant. Although the effects of hypergravity varied from cell to cell, in many cells of the wild type, hypergravity decreased the length but increased the width of the cells (Figure 3). However, hypergravity affected neither the length nor the width of the cells in the *pth2* mutant. It is indicated by these results that the *pth2* mutant could not develop short and thick hypocotyls in response to the magnitude of gravity.

Cells with longitudinal cortical microtubules were increased, while cells with transverse cortical microtubules were decreased, when hypergravity suppressed elongation growth and promoted lateral expansion in azuki bean epicotyls and Arabidopsis hypocotyls [15,16,17]. Conversely, microgravity decreased cells with longitudinal cortical microtubules, while it increased cells with transverse cortical microtubules when it promoted elongation growth and suppressed lateral expansion in Arabidopsis hypocotyls [24,25]. It is suggested by these results that regulating the orientation of cortical microtubules contributes to the modification of the body shape of plants to resist gravitational force. In this study, hypergravity affected neither the body shape nor the microtubule orientation in the *pth2* mutant (Figure 1, Figure 3 and Figure 4). The *pth2* mutant may lack the capacity to modify body shape by regulating the orientation of cortical microtubules in response to the magnitude of gravity.

The cell wall rigidity of the shoots was increased by hypergravity, while microgravity decreased the cell wall rigidity of the shoots [5,26]. It is suggested by these results that an increase in the cell wall rigidity in response to the magnitude of gravity was considered an essential component of gravity resistance, in addition to the development of a short and thick body. Hypergravity affected neither the cell wall extensibility nor the breaking load of the cell wall in the hypocotyls of the *pth2* mutant (Figure 5). Namely, cell wall rigidity in the *pth2* mutant was not affected by hypergravity. It is indicated by these results that the *pth2* mutant was not only unable to develop short and thick hypocotyls but also to increase the cell wall rigidity in response to the magnitude of gravity. The cell wall thickness was increased by hypergravity when the cell wall rigidity of shoots was increased by hypergravity [5,27]. On the other hand, the cell wall thickness was decreased by microgravity when the cell wall rigidity of the shoots was decreased by microgravity [5,27]. It is suggested by these results that the regulation of the cell wall thickness contributes to the modification of the cell wall rigidity to resist the gravitational force. In this study, neither the cell wall rigidity nor the cell wall thickness was affected by hypergravity in the *pth2* mutant (Figure 5 and Figure 6). The *pth2* mutant may be unable to modify cell wall rigidity because it cannot regulate cell wall thickness in response to the magnitude of gravity.

The *pth2* mutant had short and thick hypocotyls and higher cell wall rigidity even at 1 *g*, without any further changes in the hypocotyl growth or cell wall rigidity due to hypergravity at 300 *g* (Figure 1, Figure 3 and Figure 5). It is suggested by these results that *pth2* is the mutant hypersensitive to gravitational force and that the effects of gravity are saturated at 1 *g*. Under microgravity conditions in space, hypocotyl growth and the cell wall rigidity of the *pth2* mutant may be restored to the wild-type level, as shown in tubulin mutants [25,26]. Space experiments are needed to examine this possibility. In wild type Arabidopsis, *PTH2* may play an essential role in plant body construction in response to a wide range of gravity forces. Preliminary transcriptome analyses showed that the expression level of *PTH2* was higher under hypergravity conditions compared to 1 *g*. In contrast, the expression of *PTH2* tended to be downregulated under microgravity conditions in space. It is suggested by the results that the expression of *PTH2* is regulated by the gravitational signal. A detailed analysis of *PTH2* expression under different gravity conditions is needed. The analysis should include the lunar (0.17 *g*) and Martian levels of gravity (0.38 *g*) because plants are an essential component of the bioregenerative life-support systems required for long-term human space exploration [28,29,30]. Additionally, understanding the mechanism of gravity resistance contributes to the achievement of plant cultivation in space.

As described above, in the *pth2* mutant, multiple processes of gravity resistance do not work properly. *PTH2* encodes peptidyl-tRNA hydrolase II, an enzyme that releases tRNA from peptidyl-tRNA by cleaving the ester bond between the peptide and the tRNA [21]. It has been shown that protein biosynthesis is suppressed by the accumulation of peptidyl-tRNA. Therefore, the *pth2* mutant may not induce proper gravity resistance responses by suppressing the synthesis of proteins responsible for gravity resistance. For example, the γ-tubulin complex and katanin genes, microtubule-associated proteins required to reorient cortical microtubules, were transiently increased by hypergravity [31,32]. Thus, in the *pth2* mutant, the synthesis of proteins, such as the γ-tubulin complex and katanin, may be suppressed. However, it would be difficult to specifically suppress only the synthesis of proteins involved in gravity resistance. *PTH2* may be involved in the signal perception in the initial step of transduction in gravity resistance. If so, this might explain why multiple processes of gravity resistance do not work properly. The detailed role of *PTH2* is currently under investigation.

## 5. Conclusions

*PTH2* was identified as a new gene responsible for gravity resistance in Arabidopsis seedlings. Future studies on the function of *PTH2* may contribute to understanding the mechanism of gravity resistance and the achievement of efficient plant cultivation in space.

## Data Availability

The data presented in this study are available on request from the corresponding author upon reasonable request.

## References

[1] Kenrick P., Crane P. (1997). The origin and early evolution of plants on land. Nature.

[2] Volkmann D., Baluška F. (2006). Gravity: One of the driving forces for evolution. Protoplasma.

[3] Nakamura M., Nishimura T., Terao-Morita M. (2019). Gravity sensing and signal conversion in plant gravitropism. J. Exp. Bot..

[4] Hoson T., Soga K. (2003). New Aspects of Gravity Responses in Plant Cells. Int. Rev. Cytol..

[5] Soga K. (2013). Resistance of plants to gravitational force. J. Plant Res..

[6] Waldron K.W., Brett C.T. (1990). Effects of Extreme Acceleration on the Germination, Growth and Cell Wall Composition of Pea Epicotyls. J. Exp. Bot..

[7] Kasahara H., Shiwa M., Takeuchi Y., Yamada M. (1995). Effects of hypergravity on the elongation growth in radish and cucumber hypocotyls. J. Plant Res..

[8] Hoson T., Nishitani K., Miyamoto K., Ueda J., Kamisaka S., Yamamoto R., Masuda Y. (1996). Effects of hypergravity on growth and cell wall properties of cress hypocotyls. J. Exp. Bot..

[9] Soga K., Wakabayashi K., Hoson T., Kamisaka S. (1999). Hypergravity increases the molecular mass of xyloglucans by decreasing xyloglucan-degrading activity in azuki bean epicotyls. Plant Cell Physiol..

[10] Soga K., Harada K., Wakabayashi K., Hoson T., Kamisaka S. (1999). Increased Molecular Mass of Hemicellulosic Polysaccharides is Involved in Growth Inhibition of Maize Coleoptiles and Mesocotyls under Hypergravity Conditions. J. Plant Res..

[11] Soga K., Wakabayashi K., Hoson T., Kamisaka S. (2001). Gravitational force regulates elongation growth of arabidopsis hypocotyls by modifying xyloglucan metabolism. Adv. Space Res..

[12] Wakabayashi K., Soga K., Kamisaka S., Hoson T. (2005). Changes in levels of cell wall constituents in wheat seedlings grown under continuous hypergravity conditions. Adv. Space Res..

[13] Wasteneys G.O., Galway M.E. (2003). Remodeling the Cytoskeleton for Growth and Form: An Overview with Some New Views. Annu. Rev. Plant Biol..

[14] Baskin T.I. (2005). Anisotropic expansion of the plant cell wall. Annu. Rev. Cell Dev. Biol..

[15] Soga K., Wakabayashi K., Kamisaka S., Hoson T. (2006). Hypergravity induces reorientation of cortical microtubules and modifies growth anisotropy in azuki bean epicotyls. Planta.

[16] Matsumoto S., Kumasaki S., Soga K., Wakabayashi K., Hashimoto T., Hoson T. (2010). Gravity-induced modifications to devel-opment in hypocotyls of Arabidopsis tubulin mutants. Plant Physiol..

[17] Murakami M., Soga K., Kotake T., Kato T., Hashimoto T., Wakabayashi K., Hoson T. (2016). Roles of MAP65-1 and BPP1 in Gravity Resistance of Arabidopsis hypocotyls. Biol. Sci. Space.

[18] Skagen E.B., Iversen T.-H. (1999). Simulated weightlessness and hyper-g results in opposite effects on the regeneration of the cortical microtubule array in protoplasts from Brassica napus hypocotyls. Physiol. Plant..

[19] Nakabayashi I., Karahara I., Tamaoki D., Masuda K., Wakasugi T., Yamada K., Soga K., Hoson T., Kamisaka S. (2006). Hyper-gravity stimulus enhances primary xylem development and decreases mechanical properties of secondary cell walls in in-florescence stems of *Arabidopsis thaliana*. Ann. Bot..

[20] Mabuchi A., Soga K., Wakabayashi K., Hoson T. (2016). Phenotypic screening of Arabidopsis T-DNA insertion lines for cell wall mechanical properties revealed ANTHOCYANINLESS2, a cell wall-related gene. J. Plant Physiol..

[21] Das G., Varshney U. (2006). Peptidyl-tRNA hydrolase and its critical role in protein biosynthesis. Microbiol. Read..

[22] Hattori T., Otomi Y., Nakajima Y., Soga K., Wakabayashi K., Iida H., Hoson T. (2020). MCA1 and MCA2 Are Involved in the Response to Hypergravity in Arabidopsis Hypocotyls. Plants.

[23] DuBois M., Gilles K.A., Hamilton J.K., Rebers P.A., Smith F. (1956). Colorimetric method for determination of sugars and related substances. Anal. Chem..

[24] Soga K., Yamazaki C., Kamada M., Tanigawa N., Kasahara H., Yano S., Kojo K.H., Kutsuna N., Kato T., Hashimoto T. (2018). Modification of growth anisotropy and cortical microtubule dynamics in Arabidopsis hypocotyls grown under microgravity conditions in space. Physiol. Plant..

[25] Kato S., Murakami M., Saika R., Soga K., Wakabayashi K., Hashimoto H., Yano S., Matsumoto S., Kasahara S., Kamada M. (2022). Suppression of Cortical Microtubule Reorientation and Stimulation of Cell Elongation in Arabidopsis Hypocotyls under Microgravity Conditions in Space. Plants.

[26] Tanimura Y., Mabuchi A., Soga K., Wakabayashi K., Hashimoto H., Yano S., Matsumoto S., Kasahara H., Kamada M., Shimazu T. (2022). Suppression of secondary wall formation in the basal supporting region of Arabidopsis inflorescence stems under microgravity conditions in space. Biol. Sci. Space.

[27] Hoson T., Wakabayashi K. (2015). Role of the plant cell wall in gravity resistance. Phytochemistry.

[28] Kiss J.Z. (2014). Plant biology in reduced gravity on the Moon and Mars. Plant Biol..

[29] Medina F.J., Manzano A., Villacampa A., Ciska M., Herranz R. (2021). Understanding reduced gravity effects on early plant de-velopment before attempting life-support farming in the Moon and Mars. Front. Astron. Space Sci..

[30] Medina F.-J., Manzano A., Herranz R., Kiss J.Z. (2022). Red Light Enhances Plant Adaptation to Spaceflight and Mars *g*-Levels. Life.

[31] Soga K., Kotake T., Wakabayashi K., Kamisaka S., Hoson T. (2008). Transient increase in the transcript levels of γ-tubulin complex genes during reorientation of cortical microtubules by gravity in azuki bean (Vigna angularis) epicotyls. J. Plant Res..

[32] Soga K., Kotake T., Wakabayashi K., Kamisaka S., Hoson T. (2009). The Transcript Level of Katanin Gene is Increased Transiently in Response to Changes in Gravitational Conditions in Azuki Bean Epicotyls. Biol. Sci. Space.

